# Acupuncture on Obesity: Clinical Evidence and Possible Neuroendocrine Mechanisms

**DOI:** 10.1155/2018/6409389

**Published:** 2018-06-14

**Authors:** Kepei Zhang, Shigao Zhou, Chunyan Wang, Hanchen Xu, Li Zhang

**Affiliations:** ^1^Institute of Digestive Diseases, Longhua Hospital, Shanghai University of Traditional Chinese Medicine, Shanghai, China; ^2^Department of TCM Demonstration, Longhua Hospital, Shanghai University of Traditional Chinese Medicine, Shanghai, China

## Abstract

**Objective:**

Acupuncture, as one of the complementary and alternative medicines, represents an efficient therapeutic option for obesity control. We conducted a meta-analysis to investigate the effectiveness of acupuncture in obesity and also summarized the available studies on exploring the mechanisms.

**Design:**

We searched six databases from the inception to April 2017 without language restriction. Eligible studies consisted of acupuncture with comparative controls ((1) sham acupuncture, (2) no treatment, (3) diet and exercise, and (4) conventional medicine). The primary outcomes consisted of BMI, body weight reduction, and incidence of cardiovascular events (CVD). Secondary outcomes included waist circumference (WC), waist-to-hip ratio (WHR), body fat mass percent, body fat mass (kg), total cholesterol (TC), triglyceride (TG), glucose, low density lipoprotein cholesterol (LDL-c) reduction, high density lipoprotein cholesterol (HDL-c) increase, and adverse effects. The quality of RCTs was assessed by the Cochrane Risk of Bias Tool. Subgroup analyses were performed according to types of acupuncture. A random effects model was used to adjust for the heterogeneity of the included studies. Publication bias was assessed using funnel plots.

**Main Results:**

We included 21 studies with 1389 participants. When compared with sham acupuncture, significant reductions in BMI (*MD*=-1.22,* 95*%*CI*=-1.87 to -0.56), weight (*MD*=-1.54,* 95*%*CI*=-2.98 to -0.11), body fat mass (kg) (*MD*=-1.31,* 95*%*CI*=-2.47 to -0.16), and TC (*SMD*=-0.63,* 95*%*CI*=-1.00 to -0.25) were found. When compared with no treatment group, significant reductions of BMI (*MD*=-1.92,* 95*%*CI*=-3.04 to -0.79), WHR (*MD*=-0.05,* 95*%*CI*=-0.09 to -0.02), TC (*MD*=-0.26,* 95*%*CI*=-0.48 to -0.03), and TG (*MD*=-0.29* 95*%*CI*=-0.39 to -0.18) were found. When compared with diet and exercise group, significant reduction in BMI (*MD*=-1.24,* 95*%*CI*=-1.87 to -0.62) and weight (*MD*=-3.27* 95*%*CI*=-5.07 to -1.47) was found. Adverse effects were reported in 5 studies.

**Conclusions:**

We concluded that acupuncture is an effective treatment for obesity and inferred that neuroendocrine regulation might be involved.

## 1. Introduction

Obesity is a chronic disease characterized by the rise of body fat stores. It is caused by the interaction of genetic, dietary, lifestyle, and environment factors. The prevalence of obesity among children, adolescents, and adults has been dramatically increased during the last decades. Obesity and overweight are closely related to type 2 diabetes, hypertension, and coronary heart disease [1]. The World Health Organization (WHO) indicates that more than 1.9 billion adults, 18 years and older, were overweight. Of these, over 650 million were obese in 2016. According to a report in the JAMA Journal of Internal Medicine, more than two-thirds of people in the United States are considered to be overweight or obesity [2]. Data from China's fourth national physical fitness survey in 2014 showed that the morbidity of obesity in adult and the aged reached 10.5% and 13.9%, respectively, which showed 0.6% and 0.9% increase in comparison to the data in 2010. Moreover, the epidemics of obesity and overweight are not limited in developed countries, and the prevalence also increases among people in developing countries.

Obesity can be defined by BMI. According to the WHO definition, a BMI over 25kg/m^2^ is taken as overweight and over 30kg/m^2^ as obese. In terms of the physique of the Asia-Pacific population and the characteristics of obesity-related disease, the WHO obesity adviser group agrees that BMI over 23kg/m^2^ is defined as overweight and over 25kg/m^2^ as obese. In addition, other guidelines also include parameters such as WC and WHR to define obesity.

Numerous people cannot manage the weight only through dietary change and increasing physical activity. Although pharmaceutical treatments for obesity such as Fenfluramine and Sibutramine are effective, there exist various limits due to security reasons [3–6]. As an alternative intervention for obesity, acupuncture is relatively easy, cheap, and safe and has been widely used in clinical practice [5–8] in both China and other countries. Although efficacy of the acupuncture therapy has been reported, the underlying mechanisms have not been completely illustrated. Therefore, we conducted a systematic review and meta-analysis to evaluate the effectiveness of acupuncture in obesity and also summarized the present studies on exploring mechanisms under acupuncture treatment in obesity animals.

## 2. Methods

### 2.1. Search Strategy

To identify studies of acupuncture on obesity, retrievals were implemented in three English databases (PubMed, EMBASE, and Cochrane Library) and three Chinese databases (VIP information database, Chinese National Knowledge Infrastructure, and Wanfang Data Information Site) from the inception to April 2017. The search strategies were (weight loss OR overweight OR obesity OR weight control OR simple obesity OR weight reduction OR weight increase OR weight decrease OR weight watch OR overeat OR overfeed OR slim) AND (acupuncture and moxibustion OR acupuncture OR embedding therapy OR acupoint catgut embedding OR electro-acupuncture OR EA OR auricula-acupuncture OR ear seed pressure OR auricular plaster OR auricular acupuncture OR auricular acupressure OR fire needle OR moxibustion OR herbal acupuncture OR dermal needle OR aqua acupuncture OR body acupuncture OR meridians OR abdominal acupuncture) AND (clinical trial OR clinical study OR efficacy OR effectiveness) AND (random OR random$). Conference proceedings, dissertations, and reference lists of retrieved articles were also searched manually for additional relevant studies.

### 2.2. Inclusion and Exclusion Criteria

#### 2.2.1. Types of Studies

Published randomized controlled trials (RCTs) compared acupuncture with control (no treatment, placebo acupuncture, western medicine, diet or exercise, etc.) and assessed the efficacy of acupuncture on obesity and overweight. We excluded quasi-randomized studies, such as those allocated by using alternate days of week. No restriction was imposed on blinding. Comments, case reports, technical reports, animal studies, self-control studies, or non-RCTs were excluded. No language restriction was made for selecting the studies.

#### 2.2.2. Types of Participants

We included participants with no limitation of age and gender. All appropriate definitions of overweight or obesity including BMI, body weight, or percentage of weight excess compared with ideal weight were accepted. A diagnosis of simple obesity patients was included. The secondary obesity which was complicated with hypothalamus disease, anterior hypopituitarism, hypothyroidism, hypercriticism, hypogonadotropic hypogonadism, pregnancy, lactation, polycystic ovarian syndrome (PCOS), menstrual disorder, amenorrhea, or other serious medical conditions was excluded.

#### 2.2.3. Types of Intervention

We recruited trials with various acupuncture therapies. The acupuncture therapy included classical acupuncture, electroacupuncture (EA), laser acupuncture, catgut embedding, auricular acupressure, and auricular acupuncture, which could be analyzed in subgroup. Studies that combined acupuncture with other therapies such as medication, moxibustion, or message were excluded; the studies with lifestyle intervention such as diet and exercise in treatment group were also included. Based on the different acupuncture therapies, we performed the subgroup analysis. The control inventions were divided into four types, sham acupuncture ((1) needle inserting into skin but not penetrating the exact acupoints; (2) needle inserted into an area where it is near the exact acupoints), no treatment, diet, and exercise therapy, medicine.

#### 2.2.4. Types of Outcome Measures

The primary outcomes consisted of BMI, body weight reduction, and the incidence of CVD. Secondary outcomes included WC, WHR, body fat mass percent, body fat mass (kg), serum cholesterol (TC), triglyceride (TG), glucose, low density lipoprotein cholesterol (LDL-c) reduction, high density lipoprotein cholesterol (HDL-c) increase, and adverse effects.

### 2.3. Study Selection, Data Extraction, Management, and Analysis

According to the prespecified inclusion and exclusion criteria, two authors (Kepei Zhang and Chunyan Wang) separately identified the eligible studies by reading the title, abstract, and full text of every paper and then extracted the data. A discussion with the other authors was conducted to solve any discrepancies.

The following information was abstracted from all included publications: year, country, number of included patients, interventions of treatment and control groups, basic treatment, duration of treatment, adverse reactions, and outcomes. Authors of studies were contacted for clarification when necessary.

The quality of RCTs was assessed by the Cochrane Risk of Bias Tool, including seven domains: generation of a random sequence, allocation concealment, blinding of participants and personnel, blinding of outcome assessment, completeness of outcome data, selectiveness of reporting, and other biases. A score of 1 or 0 was given for each item depending on the information provided by study (1, low risk of bias, the information of the domain was adequate in the text; 0, high risk of bias, the information of the domain was inadequate in the text). The studies with the cumulative score of at 3 or more were included in our study.

Cochrane Review Manager (RevMan 5.3) software was used for statistical analysis. Binary data were reported as risk ration (RR), and continuous data were reported as mean difference (MD) when the outcomes were measured in the same way among different trials. For trials reporting the same outcome measures but used different methods, the standardized mean difference (SMD) was reported. 95% confidence interval (95%CI) was used as an effective size for the combined analysis. A random effects model was conducted to analyze pooled effects. We tested heterogeneity using the Chi^2^ statistic (with significance being set at* P*<0.1) and the* I*^2^ statistic.* I*^2^ value above 50% was set as substantial heterogeneity. Possible sources of heterogeneity were assessed by sensitivity and subgroup analysis. The existence of publication bias was checked using a funnel plot.

## 3. Results

### 3.1. Study Description and Quality Assessment

**Figure 1:**
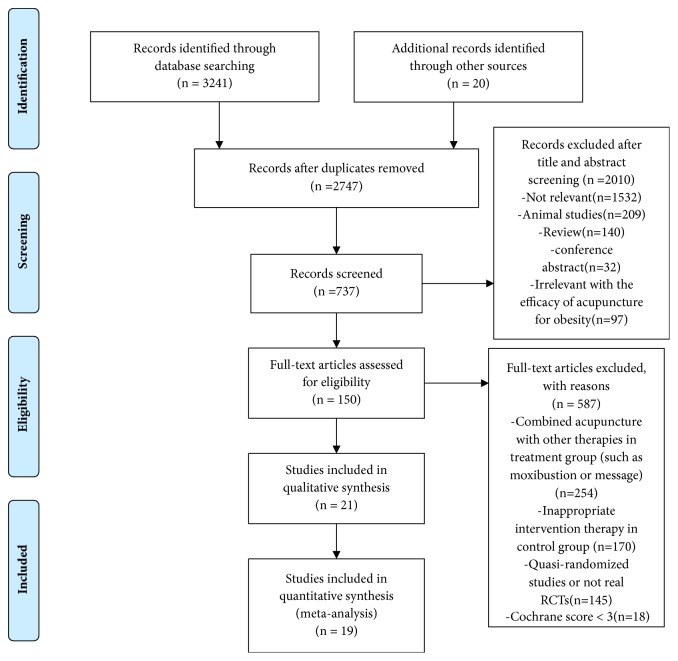
Flow diagram or the number of studies included and excluded.

A total of 3261 potentially relevant papers were retrieved. 514 duplicate records were removed. 737 articles were remaining after the scan of titles and abstract. 2010 articles (including not relevant, animal studies, review, conference abstract, and irrelevant with the efficacy of acupuncture for obesity) were excluded. 150 articles were selected after screening full-text articles. 587 articles (including studies which combined acupuncture with other therapies in treatment group, inappropriate intervention therapy in control group, quasi-randomized studies or not real RCTs, and Cochrane score<3) were excluded. Finally, we included 21 studies [9–29]: 7 [9–12, 17, 20, 25] in English and 14 [13, 16, 18–21, 21, 22, 22, 23, 23, 24, 24–26] in Chinese. 19 studies [9, 10, 12, 13, 15–29] were included in meta-analysis. The screening process is summarized in**Figure 1**. The sample size of the included studies ranged from 9 to 43, enrolling a total of 1389 participants altogether, 760 patients in the treatment group and 629 patients as the control. Meanwhile, among these studies, the study by Han 2016 [13] included two independent experiments, so we divided this study into Han-1 2016 and Han-2 2016. The study by Darbandi et al. 2014 [11] included one experiment, which contributed four independent comparisons. Descriptive analysis was used in this study. The basic characteristics of studies included are summarized in**Table 1**. Of these 22 records, there were 12 records [9–12, 15, 16, 18, 21, 24–26, 29] reporting the effect of acupuncture versus sham acupuncture, 5 [13, 17, 19, 27] acupuncture versus no treatment, 4 [20, 22, 23, 29] acupunctures versus diet and exercise, and 1 [14] acupuncture versus medicine.

The quality assessment of the included studies is summarized in**Table 2**. The majority of studies included had more or less methodological weakness according to the quality criteria applied. Of the 22 records, 1 [20] fulfilled three, 11 [9–12, 15, 17–19, 23, 24, 28] fulfilled four, 4 [14, 22, 25, 28] fulfilled five, 3 [16, 21, 27] fulfilled six, and 3 [13, 26] fulfilled seven. All records had random allocation, 14 [9–11, 13, 15, 18, 20–28] in used random number table, 4 [14, 17, 19, 29] employed draw lots, 1 [12] used urn randomization, 1 [16] performed permuted block randomization, and 1 [20] randomly listed names and assigned them to three groups. Moreover, 10 [11, 13, 15, 16, 18, 21, 24–26] mentioned blinding of participants and personnel and 7 [13, 14, 21, 26, 27] mentioned blinding of outcomes. In addition, 8 [13, 16, 22, 24–28] reported the plan of allocation and concealment. Two [20, 28] had no information about withdraws but provided complete outcome data. Three [15, 24, 25] had a loss to follow-up more than 15%.

### 3.2. Effectiveness

#### 3.2.1. Acupuncture versus Sham Acupuncture

**Figure 2:**
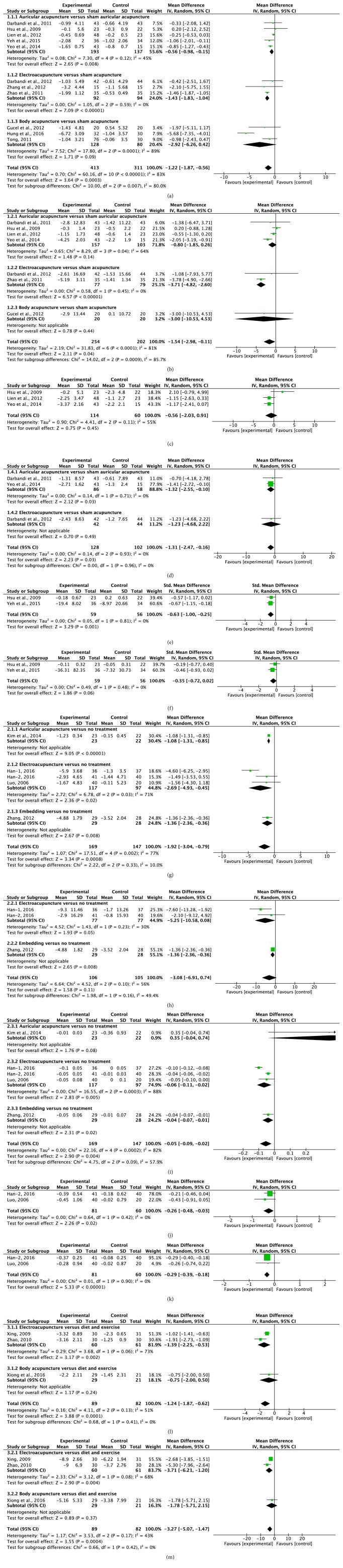
The forest plots of the efficacy of acupuncture for obesity. (1) Acupuncture versus sham acupuncture: (a) analysis of BMI in obesity patients; (b) analysis of weight loss in obesity patients; (c) analysis of WC in obesity patients; (d) analysis of body fat mass (kg) in obesity patients; (e) analysis of TC in obesity patients; and (f) analysis of TG in obesity patients. (2) Acupuncture versus no treatment: (g) analysis of BMI in obesity patients; (h) analysis of weight loss in obesity patients; (i) analysis of WHR in obesity patients; (j) analysis of TC in obesity patients and (k) analysis of TG in obesity patients. (3) Acupuncture versus diet and exercise: (l) analysis of BMI in obesity patients and (m) analysis of weight loss in obesity patients.

A total of 12 records [9–12, 15, 16, 18, 21, 24–26, 29] showed significant difference in BMI reduction between the acupuncture and sham acupuncture. The random effects model was used (*MD*=-1.22,* 95*%*CI*=-1.87 to -0.56); the high heterogeneity was detected (heterogeneity:* Chi*^2^ =60.16,* df* =10* (P*<0.00001);* I*^2^=83%). Sensitivity analysis was conducted to explore potential source of heterogeneity, which yielded* I*^2^⩾50% results after the omission of each individual study. Subgroup analyses were performed based on different acupuncture therapies. Results showed that both auricular acupuncture and EA significantly reduced BMI compared with control group (*MD*=-0.56,* 95*%*CI*=-0.98 to -0.15;* MD*=-1.43,* 95*%*CI*=-1.83 to -1.04;**Figure 2(a)**). The funnel plots were bilateral asymmetry, suggesting the publication bias may exist. A total of 7 records [9, 10, 12, 15, 18, 25, 29] reported the data of weight loss. There was significant difference between two groups. The random effects model was used (*MD*=-1.54,* 95*%*CI*=-2.98 to -0.11); high heterogeneity was detected (heterogeneity:* Chi*^2^ =31.83,* df* =6* (P*<0.00001);* I*^2^=81%). Subgroup analyses showed that EA significantly reduced weight compared with control group (*MD*=-3.71,* 95*%*CI*=-4.82 to -2.60;**Figure 2(b)**). Two records [16, 18] showed no difference in WHR loss between two groups. One record [26] showed that EA significantly reduced WHR compared with control group. Three records [15, 18, 25] showed no difference in WC loss between two groups. The random effects model was used (*MD*=-0.56,* 95*%*CI*=-2.03 to 0.91;**Figure 2(c)**); high heterogeneity was detected (heterogeneity:* Chi*^2^ =4.41,* df* =2* (P*=0.11);* I*^2^=55%). One record [11] showed auricular acupuncture and EA significantly reduced WC compared with control group. Three records [9, 10, 25] showed that the acupuncture significantly reduced body fat mass (kg). The random effects model was used (*MD*=-1.31,* 95*%*CI*=-2.47 to -0.16; heterogeneity:* Chi*^2^ =0.14,* df* =2* (P*=0.93);* I*^2^=0%). Subgroup analyses showed that auricular acupuncture significantly reduced body fat mass (kg) compared with control group (*MD*=-1.32,* 95*%*CI*=-2.55 to -0.10;**Figure 2(d)**). Three records [16, 25, 26] showed no significant difference between two groups in body fat mass percentage reduction. Two records [15, 24] showed that the auricular acupuncture significantly reduced serum total cholesterol (TC) in patients. The random effects model was used (*SMD*=-0.63,* 95*%*CI*=-1.00 to -0.25; heterogeneity:* Chi*^2^ =0.05,* df* =1* (P*=0.81);* I*^2^=0%;**Figure 2(e)**). Two records [15, 24] showed no significant difference in reducing TG in patients between groups. The random effects model was used (*SMD*=-0.35,* 95*%*CI*=-0.72 to 0.02; heterogeneity:* Chi*^2^ =0.49,* df* =1* (P*=0.48);* I*^2^=0%;**Figure 2(f)**). Two records [15, 21] showed no significant difference in reducing glucose in patients. One record [15] showed no significant difference in HDL-c and LDL-c between two groups. One [26] case of bleeding after treatment was reported in 1 record. A few participants developed minor inflammation and had mild tenderness at the acupuncture sites during the treatment in 1 record [15]; no major adverse effects were seen during the study. A subject in treatment group experienced dizziness immediately after auricular acupuncture in 1 record [18]. Slight bleeding was observed in 1 record [27].

#### 3.2.2. Acupuncture versus No Treatment

**Figure 3:**
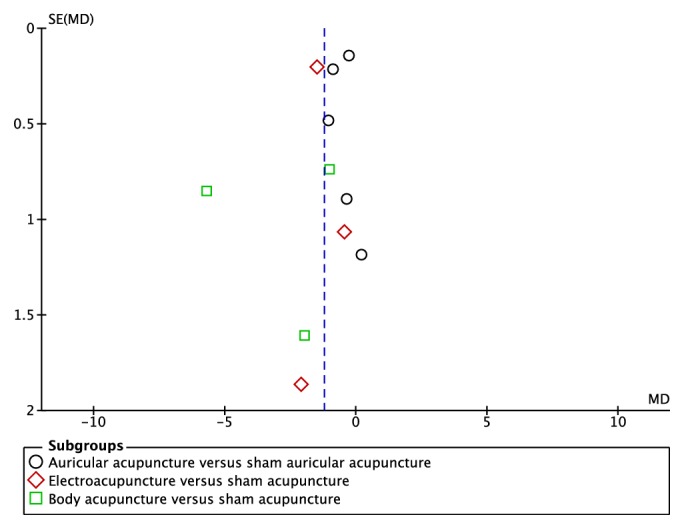
The funnel plots of the efficacy of acupuncture for obesity. Funnel plots of the effect of acupuncture on BMI between acupuncture and sham acupuncture.

Five records [13, 17, 19, 27] showed acupuncture significantly reduced BMI. The random effects model was used (*MD*=-1.92,* 95*%*CI*=-3.04 to -0.79); high heterogeneity in the data was detected (heterogeneity:* Chi*^2^ =17.51,* df* =4* (P*=0.002);* I*^2^=77%). Subgroup analyses showed that EA significantly reduced BMI compared with control group (*MD*=-2.69,* 95*%*CI*=-4.93 to -0.45;** Figures 2(g) and 3**). Three records [13, 27] reported the data of weight loss. There was no difference between two groups. The random effects model was used (*MD*=-3.08* 95*%*CI*=-6.91 to 0.74); heterogeneity in the data was detected (heterogeneity:* Chi*^2^ =4.52,* df* =2* (P*=0.10);* I*^2^=56%). Subgroup analyses showed that there was no difference between EA and control group in weight loss (*MD*=-5.25,* 95*%*CI*=-10.58 to 0.08;**Figure 2(h)**). Five records [13, 17, 19, 27] showed acupuncture significantly reduced WHR. The random effects model was used (*MD*=-0.05,* 95*%*CI*=-0.09 to -0.02); high heterogeneity in the data was detected (heterogeneity:* Chi*^2^ =22.16,* df* =4* (P*=0.0002);* I*^2^=82%). Subgroup analyses showed that EA significantly reduced WHR compared with control group (*MD*=-0.06,* 95*%*CI*=-0.11 to -0.02;**Figure 2(i)**). One record [27] showed acupuncture significantly reduced WC compared with control group. Two records [13, 19] showed the acupuncture significantly reduced TC. The random effects model was used (*MD*=-0.26,* 95*%*CI*=-0.48 to -0.03; heterogeneity:* Chi*^2^ =0.64,* df* =1* (P*=0.42);* I*^2^=0%;**Figure 2(j)**). Two records [13, 19] showed the acupuncture significantly reduced TG. The random effects model was used (*MD*=-0.29* 95*%*CI*=-0.39 to -0.18; heterogeneity:* Chi*^2^ =0.01,* df* =1* (P*=0.90);* I*^2^=0%;**Figure 2(k)**). One record [19] showed the acupuncture significantly changed LDL-c in patients. One record [19] showed no significant difference in HDL-c between two groups. Subcutaneous bleeding or hematoma after treatment was reported in 2 records [13].

#### 3.2.3. Acupuncture versus Diet and Exercise

The efficacy of acupuncture was compared to diet and exercise in 4 records [20, 22, 23, 29]. The pooled effect on BMI outcome in 3 records [22, 23, 29] showed no significant difference in BMI decrease. The random effects model was used (*MD*=-1.24,* 95*%*CI*=-1.87 to -0.62); heterogeneity was detected (heterogeneity:* Chi*^2^ =4.11,* df* =2* (P*=0.13);* I*^2^=51%). Subgroup analyses showed that EA significantly reduced BMI compared with control group (*MD*=-1.39,* 95*%*CI*=-2.25 to -0.53;**Figure 2(l)**). One record [20] reported BMI, but the data of control group was not given. Therefore, description analysis was used and it suggested that no significant difference was found between the two groups. Three records [22, 23, 29] showed the acupuncture significantly reduced weight. The random effects model was used (*MD*=-3.27* 95*%*CI*=-5.07 to -1.47); heterogeneity was detected (heterogeneity:* Chi*^2^ =3.53,* df* = 2* (P*=0.17);* I*^2^=43%). Subgroup analyses showed that EA significantly reduced body weight compared with control group (*MD*=-3.71,* 95*%*CI*=-6.21 to -1.20;**Figure 2(m)**). One record [22] showed the acupuncture significantly changed WHR in the first duration of treatment, but no significant difference was found in the last two durations between two groups. One record [29] showed the treatment group was more effective than the control group in WC loss. No significant difference in body fat mass (kg) was found between two groups in 1 record [29]. One record [22] showed no significant change between two groups in serum TC, TG, LDL-c, and HDL-c.

#### 3.2.4. Acupuncture versus Medicine

Acupuncture was compared to medicine in 1 record [14]. There was no significantly different between two groups in BMI and weight decrease. Control group was more effective than the acupuncture group in WC and WHR reduction.

### 3.3. Possible Mechanisms of Acupuncture on Obesity

**Figure 4:**
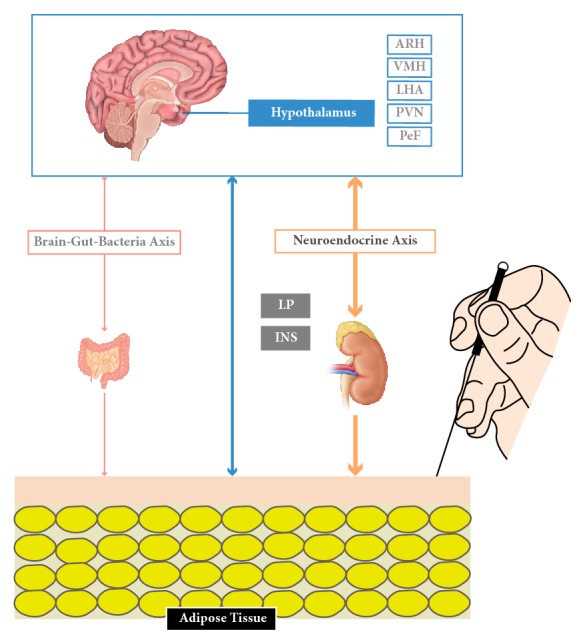
The potential neuroendocrine regulation under the efficacy of acupuncture in the animal studies.

Acupuncture is believed to be involved in neuroendocrine axis regulation. Modulating eating habits and energy metabolism are the promising strategies for obesity, and it is an immensely complex process involving the gastrointestinal tract, many hormones, and both the central and autonomic nervous systems (**Figure 4**).

The arcuate nucleus of the hypothalamus (ARH) is the main regulatory organ for appetite in human. In diet-induced obesity (DIO) rats, EA treatment significantly decreased food intake and reduced body weight compared with the untreated rats. Further analysis revealed that EA treatment increased peptide levels of *α*-MSH and mRNA expression of its precursor proopiomelanocortin (POMC) in ARH neurons. In addition, *α*-MSH in cerebral spinal fluid (CSF) elevated upon EA application. However, the lesion in ARH could abolish the inhibition effect of EA on food intake and body weight, suggesting the beneficial effects of EA treatment are acted through ARH, and that the stimulation of *α*-MSH expression and release might be involved in the process [30]. In 14-week high-fat diet feeding rats, 4-week EA treatment causes a reduction of both in body weight and energy intake, along with the upregulation of the cocaine and amphetamine-regulated transcript (CART) peptide, an anorexigenic peptide in the arcuate nucleus (ARC) [31].

Activating the satiety center tends to be one of the effective methods in preventing obesity. Su et al. [32] have shown that acupuncture can raise the frequency of neural discharge in the hypothalamic ventral medial nucleus (VMH), indicating acupuncture could improve the excitability of the medial nucleus in experimental obese animals. Liu et al. [33] have found that the frequency of spontaneous discharges of nerve cells in VMH and the levels of tyrosine (Tyr), dopamine (DA), tryptophan (Typ), and 5-hydroxytryptamine (5-HT)/5-hydroxyindole acetic acid (5-H1AA) ratio were elevated, along with the decrease of 5-HT level upon 12 days of consecutive acupuncture treatment. Lateral hypothalamic area (LHA) is the main neuroregulator in triggering ingestion. Acupuncture is reported to reduce excitation of LHA, inhibit hyperorexia, and regulate the activity of 5-HT, the catecholamine neurotransmitter, and ATPase activity in the LHA [34, 35].

Some studies [36, 37] believed that acupuncture could improve the frequency of spontaneous discharges of nerve cell in the paraventricular nucleus (PVN) and reduced the activity of hypothalamic perifornical nucleus (PeF) neurons. Ji et al. [38] concluded that an upregulation of anorexigenic factor POMC production in the nucleus tractus solitarius (NTS) and hypoglossal nucleus (HN) regions were generated by EA Zusanli (ST36), thus preventing food intake and causing weight loss. Signal transduction of EA stimuli included expression of transient receptor potential vanilloid type-1 (TRPV1) and neuronal nitric oxide synthase (nNOS) in the ST36 and the NTS/gracile nucleus through somatosensory afferents-medulla pathways. Kim et al. [39] found that stimulation of auricular acupuncture point affected the expression of NPY expression in the ARN and PVN in rats. Fu et al. [40] suggested that transcription factor STAT5 in the central nervous system plays different roles in the hypothalamus and white fat tissue during gene transcription, and acupuncture could regulate a large amount of differentially expressed genes toward their normal expression, especially genes in the hypothalamus. Thus, the weight loss effect of acupuncture might be attributed to its functional gene regulatory mechanisms. Upregulation the transcription of adenosine 5′-monophosphate-activated protein kinase*α*2 (AMPK*α*2), promotion protein expression of liver kinase B1 (LKB1) and AMPK*α*1, and inhibition acetyl-CoA carboxylase (ACC) protein expression in the hypothalamus were observed after 4 weeks of EA treatment [41]. However, auricular acupuncture stimulation is reported to be associated with satiation formation and preservation in the hypothalamus but fails to work on anorexia activity [42].

Certain hormones including insulin and ghrelin may influence appetite in the hypothalamus [43]. One study [44] showed that downregulation of ghrelin in the stomach and neuropeptide Y (NPY) in the hypothalamus was in line with the reduction in food intake in rats receiving EA stimulation once every day. Liu et al. [45] speculated that, in high-fat diet (HFD) animals, EA treatment (ST36 and LI11, 20 minutes per day for 28 days) could reduce the body weight, homeostasis model assessment-insulin resistance index, adipocyte diameters, and neuroprotein Y/agouti-related protein and protein tyrosine phosphatase 1B levels. In db/db mice, Liang et al. [46] found that EA treatment (five times per week for eight weeks) contracted the increase of fasting blood glucose, food intake, and body mass and maintained insulin levels via stimulation of skeletal muscle Sirtuin 1 (SIRT1)/peroxisome proliferator-activated receptor *γ* coactivator 1*α* (PGC-1*α*), suggesting the role of EA in improving insulin resistance. Gong et al. [47] applied EA stimulation to diet-induced obese rats for four weeks and observed the reduced body weight, plasma levels of leptin, and increased expression of leptin receptor in the hypothalamus. In addition, Shen et al. [48] discovered that four weeks of EA treatment caused remodeling white adipose tissues (WAT) to brown adipose tissue (BAT) via inducing uncoupling protein-1 (UCP1) in EA group. Besides, acupuncture also adjusted the intestinal flora, achieving the balance of brain-gut-bacteria axis [49–51].

## 4. Discussion

Here we selected 21 RCTs including 1389 patients suffering from obesity to evaluate the efficacy of acupuncture. We found that acupuncture was more effective than shame acupuncture in BMI, weight, body fat mass (kg), and TC; acupuncture was more effective than no treatment group in BMI, WHR, and TG. In addition, acupuncture is showed to be more effective than diet and exercise group in BMI and weight loss. To a limited extent, we concluded that acupuncture is an effective treatment for obesity.

Currently, the etiology of obesity has not been defined yet; many factors such as neuromodulation, viral, immune, endocrine, free radical, and genetics are reported to be involved [52–57]. Each indicator can affect a wide range of factors, hormones, and even genetic changes, so the mechanisms of acupuncture on obesity tend to be the simultaneous adjustment of multiple systems and targets. So, we reviewed the potential mechanisms under the efficacy of the animal studies and highlighted neuroendocrine regulation to be essential in the process.

The limitations of this work are as follows:

(1) Bias risk exists because most studies do not describe the allocation of hidden methods or use blind methods [58], which might result in performance bias and detection bias.

(2) The number of samples of each trial is relatively small, which might cause the insufficient sample size for analysis and test efficacy.

(3) The researchers are evaluated by different diagnostic criteria; inclusion and exclusion criteria, forms of acupuncture (acupuncture, EA, ear needles, ear pressure, and embedding), the course of treatment (different acupoints, duration of treatment), basic intervention (diet and exercise), and the confounding factors are different, which might increase heterogeneity.

(4) Some researchers do not mention the methods used in dealing with missing data although they have a loss to follow-up more than 15%. Most studies lack following course and fail to understand the long-term effects of auricular acupressure treatment, which might increase attrition bias.

(5) There are some objective factors like language and limited search resource, which may lead to the incomplete searching.

In conclusion, acupuncture is a reasonable and effective treatment for people who suffer from obesity. However, according to CONSORT Declaration and STRICTA Standard, some researchers point out that the efficacy of acupuncture on mild obesity is not significant, which is difficult for readers to understand the rationality of the study design, the correctness of the implementation, the authenticity of the results, and the clinical applications [59]. Obesity is a chronic condition, requiring long-term treatment. However, the treatment period of obesity is rather short in many studies, varying from 3-8 weeks. Follow-ups are needed to observe curative effect since the bodyweight might be easily rebound. In clinical, obesity is the major risk of cardiovascular events, so we put the incidence of CVD as the primary outcome; however, no included studies set CVD as primary outcome. More researches need to be done to evaluate CVD as the curative effect of acupuncture treatment for obesity in clinical. This systematic review was conducted to critically assess evidence from RCTs regarding the efficacy of various types of acupuncture therapies on obesity. We analyzed the outcomes which were related to obesity comprehensively. In the future, larger number of samples and higher-quality randomized controlled trials are required to verify the clinical effectiveness in treating obesity by acupuncture. Moreover, understanding the mechanisms under the efficacy of acupuncture on weight loss provides reliable experimental basis, thus convincing the patients with obesity with the application of acupuncture.

## Figures and Tables

**Table 1:** Characteristics of the included studies.

**Included studies**	**Country**	**Number of participants** **Trial Control**		**Interventions Trial Control**		**Basic treatment**	**Duration of treatment** **(week)**	**Adverse reactions**	**Outcomes**
**Darbandi** **et al., 2012 [9]**	Iran	43	43	Auricular acupressure	Sham auricular	Low-calorie diet	6	None	BW, BMI, BFM, plasma leptin

**Darbandi** **et al., 2013 [10]**	Iran	42	44	Electro-acupuncture	Sham acupuncture	Low-calorie diet	6	None	BM, BMI, BFM, plasma leptin

**Darbandi** **et al., 2014 [11]**	Iran	20/20	20/20	Body electro-acupuncture/ Auricular acupuncture	Sham body electro-acupuncture/ sham Auricular acupuncture	Low-calorie diet	6	None	Height, WHR, BMI, trunk fat mass, Cr, Albumin, Uric acid, FBS, HLD-c, LDL-c, WBC, RBC

**Gucel** **et al., 2012 [12]**	Turkey	20	20	Body acupuncture	Sham acupuncture	None	5	Not reported	Weight, BMI, insulin, leptin, ghrelin, cholecystokinin

**Han-1, 2016 [13]**	China	36	37	Body electro-acupuncture	No treatment	Dietary and exercise	4	2 Subcutaneous bleeding and hematoma	BMI, WC, hip circumference, WHR and spleen dampness improve the situation of symptom score

**Han-2, 2016 [13]**	China	40	41	Body electro-acupuncture	No treatment	Dietary and exercise	4	3 Subcutaneous bleeding and hematoma	BMI, WC, hip circumference, WHR and spleen dampness improve the situation of symptom score

**He** **et al., 2008 [14]**	China	40	40	Body electro-acupuncture + Auricular acupuncture	Sibutramine	None	8	No reported	Body weight, BMI, waist and hip circumference and WHR

**Hsu** **et al., 2009 [15]**	Taiwan	23	22	Auricular acupuncture	Shame auricular acupuncture	None	6	1 Minor inflammation, 8 mild tenderness	Body weight, BMI, WC, obesity-related hormone peptides

**Hung** **et al., 2016 [16]**	Taiwan	32	30	Verum laser acupuncture	Sham laser acupuncture	None	3	None	BMI, BFP, waist-to-buttock ratio

**Kim** **et al., 2014 [17]**	Korea	25	24	Auricular acupressure	No treatment	None	4	None	Weight, BMI, body fat mass percentage, WHR

**Lien** **et al., 2012 [18]**	Taiwan	48	23	Auricular stimulation	Sham auricular acupuncture	None	8	1 dizziness	BMI, weight, obesity-related hormone peptids, life quality scores

**Luo, 2006 [19]**	China	40	20	Electro-acupuncture / only acupuncture	No treatment	None	3	Not reported	WHR, MBI, TC, TG, HDL-c, LDL-c, LEP, Adiponectin(ADI)

**Nourshahi** **et al., 2009 [20]**	Iran	9	9	Acupuncture	No treatment/diet and exercise	Diet and exercise	8	Not reported	Body weight, skin fold thickness, BMI, fat mass

**Tong, 2011 [21]**	China	76	42	Acupuncture	Placebo-acupuncture	Diet	5	None	BMI, TC, TG, Glucose, BUN, Uric Acid and adverse reactions

**Xing, 2009 [22]**	China	31	30	Electroacupuncture	Diet and exercise	Diet and exercise	8	No reported	Body weight, BMI, TG, TC, LDL-c, HDL-c, WHR, Leptin

**Xiong** **et al., 2016 [23]**	China	29	21	Acupuncture	Exercise	None	4	No reported	Body weight, BMI

**Yeh** **et al., 2015 [24]**	Taiwan	36	34	Auricular electrical stimulation +auricular acupressure	Same manner but at sham acupoints	Diet	10	No reported	BMI, blood pressure, TC, TG, Leptin, Adiponectin

**Yeo** **et al., 2014 [25]**	Korean	43	15	Ear acupuncture	Sham acupuncture	None	8	No reported	BMI, WC, weight, body fat mass (kg), percentage body fat and blood pressure

**Zhang** **et al., 2012 [26]**	China	15	15	Electro-acupuncture	Sham Electro-acupuncture	None	4	1 Bleeding	Weight, BMI, body fat mass %

**Zhang, 2012 [27]**	China	29	28	Catgut embedded	No treatment	Diet and exercise	8	The bleeding rate was 18.75% in treatment group	Weight, BMI, waistline, hipline, circumference, quality of life

**Zhao** **et al., 2011 [28]**	China	35	35	Electro-acupuncture	Sham Electro-acupuncture	None	4	No reported	BMI, Weight

**Zhao, 2010 [29]**	China	30	30	Electro-acupuncture	Diet and exercise	Diet and exercise	8	No reported	BMI, waist, FBS, 2hPG, FINS, 2hFINS

**Table 2:** Risk of bias of the included studies.

**Included studies**	**A**	**B**	**C**	**D**	**E**	**F**	**G**	**Total**

Darbandi et al., 2012 [9]	1	0	0	0	1	1	1	4

Darbandi et al., 2013 [10]	1	0	0	0	1	1	1	4

Darbandi et al., 2014 [11]	1	0	1	0	1	0	1	4

Gucel et al., 2012 [12]	1	0	0	0	1	1	1	4

Han-1, 2016 [13]	1	1	1	1	1	1	1	7

Han-2, 2016 [13]	1	1	1	1	1	1	1	7

He et al., 2008 [14]	1	0	0	1	1	1	1	5

Hsu et al., 2009 [15]	1	0	1	0	1	0	1	4

Hung et al., 2016 [16]	1	1	1	0	1	1	1	6

Kim et al., 2014 [17]	1	0	0	0	1	1	1	4

Lien et al., 2012 [18]	1	0	1	0	1	0	1	4

Luo, 2006 [19]	1	0	0	0	1	1	1	4

Nourshahi et al., 2009 [20]	1	0	0	0	0	1	1	3

Tong, 2011 [21]	1	0	1	1	1	1	1	6

Xing, 2009 [22]	1	1	0	0	1	1	1	5

Xiong et al., 2016 [23]	1	0	0	0	1	1	1	4

Yeh et al., 2015 [24]	1	1	1	0	0	0	1	4

Yeo et al., 2014 [25]	1	1	1	0	1	0	1	5

Zhang et al., 2012 [26]	1	1	1	1	1	1	1	7

Zhang, 2012 [27]	1	1	0	1	1	1	1	6

Zhao et al., 2011 [28]	1	0	0	1	1	1	1	5

Zhao, 2010 [29]	1	0	0	0	1	1	1	4

Note: A, adequate sequence generation; B, concealment of allocation; C, blinding of participants and personnel; D, blinding of outcome assessment; E, incomplete outcome data; F, selective reporting; G, other bias; 1, low risk of bias, the information of the domain was adequate in the text; 0, high risk of bias, the information of the domain was inadequate in the text.
